# An Introduction to Simple Saccharides and Oligosaccharides, and a Decadal Review of Their Analysis in Food by Ion Chromatography and Ion Chromatography/Mass Spectrometry

**DOI:** 10.1002/fsn3.70855

**Published:** 2025-09-26

**Authors:** Hans S. A. Yates, James. F. Carter, Achamma Joseph, Melody Chaussende, Mary T. Fletcher, Viviene Santiago, Natasha L. Hungerford

**Affiliations:** ^1^ Queensland Alliance for Agriculture and Food Innovation (QAAFI) The University of Queensland, Health and Food Sciences Precinct Coopers Plains Queensland Australia; ^2^ Queensland Health Forensic and Scientific Services (QHFSS) Health and Food Sciences Precinct Coopers Plains Queensland Australia; ^3^ Townsville Community Health Services Kirwan Health Campus, Queensland Health Townsville Queensland Australia; ^4^ The Queen Elizabeth II Jubilee Hospital Metro South Hospital and Health Service Coopers Plains Queensland Australia; ^5^ School of Chemistry and Molecular Biosciences The University of Queensland St Lucia Queensland Australia

**Keywords:** health benefits, ion chromatography‐mass spectrometry (IC‐MS), pulsed amperometric detection (PAD), saccharide analysis, simple saccharides

## Abstract

This paper provides a decadal review of literature describing the analysis of simple saccharides and oligosaccharides in food by ion chromatography pulsed amperometric detection (IC/PAD) and ion chromatography mass spectrometry (IC/MS) as emerging techniques. The concentrations of these saccharides in food have important health implications and affect technical attributes of food quality. Saccharides have been analyzed over many decades using a diverse range of physical, chemical, and chromatographic techniques, each with their own advantages and disadvantages. In the past decade, ion chromatography (IC) has appeared as a contender with substantial benefits over established techniques. It is conducted without sample derivatization, unlike other techniques of comparable accuracy. Limits of detection (LOD) are also comparable, typically 20 μg/L when coupled with a pulsed amperometric detector or 1 mg/kg food using ion chromatography single quadrupole mass spectrometry. This compares to LODs of 2 g/kg food by high performance/liquid chromatography refractive index detector and 2 mg/kg food for gas chromatography/mass spectrometry. The main strength of ion chromatography is the specificity/differentiation of simple saccharides, that has resulted in the discoveries of several unreported saccharides in foods. Examples include isomaltose in flour, erlose in stingless bee honey and arabinose, ribose, rhamnose, xylose, mannose, trehalose, maltose and raffinose in paprika. Evolving future advantages of ion chromatography in saccharide analysis include the increasing utilization of mass spectrometry, more work towards a standardized analytical methodology, and the need to investigate new, novel and admixed food types so that instrumentation continues to demonstrate relevance at the cutting edge of food research.

## Graphical abstract

Graphical Abstract of Factors Affecting the Saccharides Content of Fruits and Vegetables, for Analysis

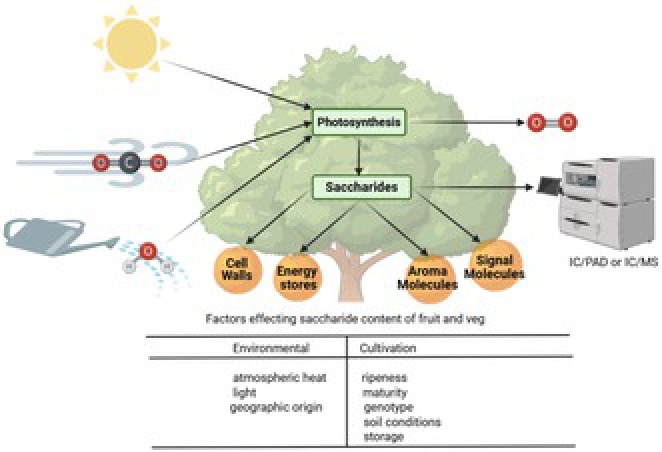

## 1 Introduction to Simple Saccharides

Saccharides, derived from the Latin *saccharum* or “sweet sand,” are considered the most abundant class of organic molecules on earth (Kurzyna‐Szklarek et al. 2022; Nouara et al. 2019) and can be divided into four main groups depending on the number of repeating units they contain. The simplest, monosaccharides, contain between three and nine carbon atoms (Kurzyna‐Szklarek et al. 2022), generally with the empirical formula (CH_2_O)_n_ (hence the name “carbohydrate”) that cannot be hydrolysed into smaller molecules. These basic units combine to form more complex saccharides that are categorized according to their degree of polymerization (DP) ranging from DP 2 to DP 10+ (Ellingson et al. 2016). DP 2 compounds are disaccharides (two joined monosaccharides), DP 3–10 compounds are generally referred to as oligosaccharides, and longer chain compounds (DP 10+) are known as polysaccharides (Mantovani et al. 2018). Oligo‐ and polysaccharides are combined in different lengths of polymerization and spatial configurations (linear and branched) to form starches, dietary fibers, gums, prebiotics, and other carbohydrates found in foods. Saccharides contribute several technical characteristics to food, the most obvious being a positive correlation with the perceived sweetness of the food and the amount of simple saccharides present.

As one of the most important classes of biochemicals, saccharides have a centralized role in the metabolism of every living organism (Meyer et al. 2022). Both over‐ and underconsumption of saccharides have known adverse health outcomes for the consumer. Additionally, there are specific population groups with metabolic conditions that are affected by the consumption of certain DP 1 and DP 2 saccharides. Examples of these include hereditary fructose intolerance, galactosemia (galactose intolerance) and lactose malabsorption (Fels and Bunzel 2022). Among these conditions, lactose malabsorption is the most widespread, with one meta‐analysis showing that 67% of the global population has this condition (Storhaug et al. 2017).

Despite the serious health effects associated with the consumption of saccharides, problems surround the accuracy of the analysis of saccharides in food. The main issues are (a) selective separation of saccharides, with most common analytical techniques having limited specificity and (b) questionable accuracy when separating and quantitating mixtures. Because of its highly selective separation of saccharides, ion chromatography with pulsed amperometric detection (IC/PAD) has been increasingly used for the analysis of saccharides in food (Malacarne et al. 2016; Vennard et al. 2020). The technique is capable of analyzing saccharides up to DP 80 (Eggleston et al. 2016) while maintaining picomole sensitivity (Zhang, Zhu, et al. 2022).

This decadal review on saccharide analysis in food by IC/PAD and ion chromatography/mass spectrometry (IC/MS) will focus more on DP 1 to DP 3 saccharides (mono‐, di‐ and tri‐saccharides), which are synonymous with the term “sugar.” DP 3 and higher are not classed as “sugars” for regulatory purposes (FAO 2021), however, as DP 3 sugars have similar nutritional characteristics to DP 1‐2 sugars (Ellingson et al. 2016), they have been included in this review. Larger saccharides (DP 3+) will be addressed briefly but have been reviewed within the last decade by Yang et al. (2020) as food polysaccharides, Mechelke et al. (2017) and Catenza and Donkor (2021) as food oligosaccharides.

### 1.1 The Chemical Complexities of Simple Saccharides

The spatial configurations of saccharide molecules are complex as they contain multiple asymmetric centres, which allow for the formation of numerous chiral compounds including enantiomers (mirror image isomers), diastereomers (non‐mirror image isomers, usually with respect to two or more chiral carbons) and epimers (diastereomeric with respect to only one chiral carbon) (Kurzyna‐Szklarek et al. 2022). In addition, saccharides can be either a ketone (ketose) in which the carbonyl group is internal to the molecule or an aldehyde (aldose) in which the carbonyl group is terminal to the molecule, as shown in Table 1. The naming convention for these configurations is the suffix “‐ulose” for ketoses or “‐ose” for aldoses. This nomenclature is, however, not used for saccharides discovered before the establishment of the naming convention, for example, fructose (or laevulose). The full systematic nomenclature of carbohydrates is described by the IUPAC‐IUBMB Joint Commission on Biochemical Nomenclature (JCBN) (McNaught 1996).

**TABLE 1:** Some examples of monosaccharides mentioned in this review and their properties. Hydrogen atoms (H) as seen in straight chain structures, have been omitted from cyclic structures for purposes of illustrative clarity (Dubovski et al. 2022; Qi and Tester 2019). (Created with BioRender.com).

Property	d‐fructose	d‐galactose	d‐glucose	l‐glucose	d‐xylose	d‐mannose
Structure: Straight chain (Fischer projection)	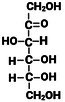	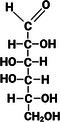	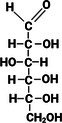	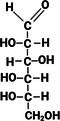	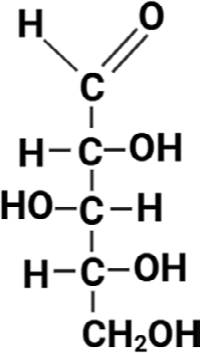	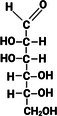
Example cyclic structure: (Haworth projection)	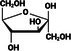	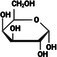	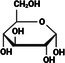	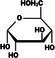	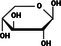	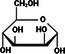
Compound class	Ketose	Aldose	Aldose	Aldose	Aldose	Aldose
Molecular formula and mass (g/mol)	C_6_H_12_O_6_ 180.2	C_6_H_12_O_6_ 180.2	C_6_H_12_O_6_ 180.2	C_6_H_12_O_6_ 180.2	C_5_H_10_O_5_ 150.1	C_6_H_12_O_6_ 180.2
Sweetness perception Index	1.73	0.32	0.74	0.74	0.61	0.40
Glycaemic Index (GI)	32	< 46	100	0	< 52	< 100

**FIGURE 1:**
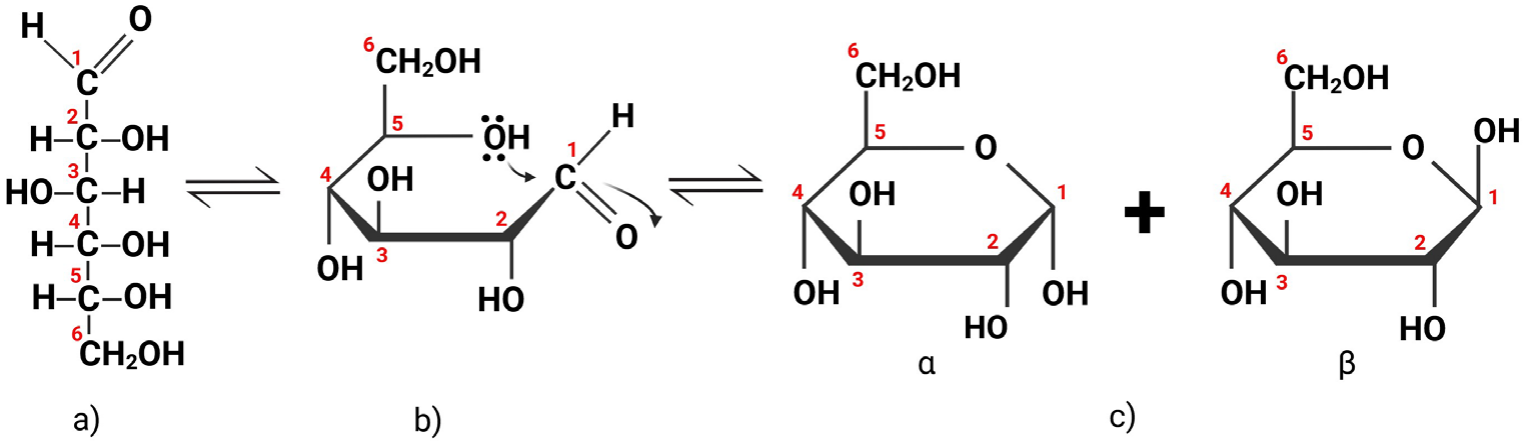
Example of straight chain and ring structures of D‐glucose: (a) open chain glucose, (b) three‐dimensional orientation allowing ring closure, (c) ring structure of D‐glucose with both α or β anomers shown. Hydrogen atoms (H) not in utilized steps (b) & (c) of this reaction have been omitted for the purposes of illustrative clarity (Created with BioRender.com).

Monosaccharides which contain five or more carbon atoms commonly form a ring structure in aqueous solutions and exist primarily in this form. This involves the C1/C2 carbonyl group reacting with the hydroxyl group at C5 to form a new bond as part of a hemiacetal/hemiketal, resulting in the most favorable ring size (Figure 1). The newly formed chiral carbon (C1 in Figure 1c) is referred to as the “anomeric carbon” and can have two orientations (*α* or *β* anomers). This process, referred to as “tautomerization,” is an equilibrium reaction between the aldose/ketose forms and the cyclic hemiacetal/hemiketal structures. The rate at which this equilibrium occurs is relatively fast. Examples of monosaccharide isomers are presented in Table 1. d‐glucose and l‐glucose are enantiomers, galactose is an epimer and diasteromer of glucose, and fructose is the ketose form of the aldose, glucose. These chemical differences give rise to different physical and technical properties, as displayed in Table 1.

The term “reducing saccharides,” broadly refers to any saccharide able to act as a reducing agent in aqueous solution. This occurs as aldose hemiacetals tautomerize to the ring‐opened aldehyde which then acts as a reducing agent. Ketose sugars are also reducing saccharides as they can convert to the equivalent aldose and the resulting aldehyde becomes oxidized (Pratt and Cornely 2014). In contrast, acetals (or ketals), formed by the reaction of the anomeric carbon with a hydroxyl of another alcohol, cannot ring open, hence no reduction reaction can take place, as is the case for sucrose (Pratt and Cornely 2014).

For DP 2 and larger saccharides, bonding between two monosaccharide units may occur via any of the hydroxyl groups of the first monosaccharide (C1–C6 in Figure 1) and the leading anomeric carbon of another molecule, via condensation (water formation). Referred to as a “glycosidic bond,” the bond can be either oriented α (bond orientation for both the terminal and anomeric C‐O are on opposite sides or *trans* orientation across the ring) or β (bonds on same side or *cis* orientation across the ring). For a disaccharide consisting only of glucose and fructose, such as sucrose, there are over 12 possible isomeric configurations (not including d‐ or l‐ forms) in nature.

### 1.2 Origins of Simple Saccharides in Food

From a biological perspective, all saccharides originate as the primary products of photosynthesis (Hassid 1969). These are then used as building blocks for more diverse and complex structures including plant cell walls, energy stores, aroma compounds and signaling molecules (Akšić, Gašić, et al. 2019; Seigler 1998). The concentrations of saccharides present in plants, vegetables, and fruits vary with growth conditions including environmental factors such as atmospheric heat (Mudrić et al. 2017), light and geographic origin (Akšić, Tosti, et al. 2019) as well as, cultivation factors for example, ripeness (Xiao et al. 2022), maturity (Sadeghi et al. 2022), genotype, soil conditions and storage (Akšić, Tosti, et al. 2019). The compositions of saccharides within plants, vegetables and fruits will also change post‐harvest if climacteric (i.e., ripening processes continues) (Marques et al. 2022). Carbohydrates, as a food source, provide energy to herbivores and omnivores and can be attached to proteins, lipids and other biomolecules, such as flavonoids (Mudrić et al. 2017).

### 1.3 Health Implications of Saccharides

For humans, carbohydrates provide 40%–80% of our dietary energy (Acunha et al. 2016; Zhang et al. 2023). Naturally occurring and added sugars (total sugars) constitute the largest proportion of this figure. Total sugars are consumed in varying quantities depending on the age group, between 13% for adults and 38% for infants in terms of total energy intake (Meyer et al. 2022). Milk, fruit, vegetables, and legumes provide naturally occurring sugars along with other important nutrients. In contrast, food with added sugars, like biscuits, cakes, and soft drinks usually do not contain mitigating beneficial nutrients. The World Health Organization (WHO) recommends that the intake of added sugars should be limited to 10% of the total energy intake for both adults and children (*Guideline*: *Sugars intake for adults and children*. *Geneva*; World Health Organization).

Greater intake of added sugars, mainly as sucrose, increases the energy density of diets with a concomitant reduction in other nutrient intake leading to weight gain and increased risk of non‐communicable diseases such as type 2 diabetes, coronary heart disease, hypertension, metabolic associated fatty liver disease, some cancers, osteoarthritis, and obstructive sleep apnoea (Guideline: Sugars intake for adults and children. Geneva; World Health Organization 2015). Apart from an increased risk of non‐communicable diseases, some people have food intolerances and irritable bowel syndromes (IBS) triggered by ingestion of naturally occurring sugars, while others may have congenital sugar disorders/deficiencies that can appear at any age.

Some examples of genetic, hereditary or dietary intolerances to specific DP 1–3 saccharides include:

- Congenital sucrase‐isomaltase deficiency (CSID), a genetic disorder that affects a person's ability to digest sucrose and maltose causing diarrhea, abdominal pain, nausea, vomiting, or reflux‐like symptoms and malabsorption of other nutrients.
- Galactosemia, an inherited metabolic disorder with an inability to break down galactose, leading to its buildup in the blood causing multiple effects including vomiting, jaundice, cataracts, and sepsis.
- Hereditary fructose intolerance, a genetic disorder in which people lack the protein or enzyme to break down fructose, leading to hypoglycemia and altered protein metabolism.
- Dietary fructose intolerance, which occurs in adulthood; is malabsorption of fructose, due to the inability of intestinal cells to break down fructose, results in increased water in the bowel lumen and rapid movement into the colon, which then ferments and leads to bloating, gas, diarrhea, and discomfort (Fedewa and Rao 2013).
- Lactose intolerance, caused by the localized bowel symptoms of diarrhea, bloating and abdominal pain.

The current solution to all these problems is to identify, avoid and/or limit these saccharides in the diet (Fels and Bunzel 2022). Malabsorption of, and intolerance to saccharides are common but poorly recognized and managed problems (Fedewa and Rao 2013). These intolerances frequently produce unexplained gastrointestinal symptoms such as abdominal bloating, gas, borborygmi, pain, distension, nausea, and diarrhea (Ferraris et al. 2018). Both malabsorption and intolerance are often unrecognized or misdiagnosed as IBS. A 2008 study estimated that one third of patients with suspected IBS had fructose malabsorption and dietary fructose intolerance (Choi et al. 2008). Patients with IBS are often prescribed a low FODMAP (fermentable oligo‐, di‐, monosaccharides, and polyols) diet (Fedewa and Rao 2013), which requires a diet low in fermentable saccharides, such as fructose, lactose, melibiose, raffinose, kestose, and stachyose (Ispiryan et al. 2019).

A 2015 consensus from the *International Carbohydrate Quality Consortium* highlighted a need for the type and amount of carbohydrate consumed (which can be described by Glycemic Index [GI] and glycemic load), to be accurately determined as the prevalence of non‐communicable diseases continues to increase (Augustin et al. 2015). GI is a numerical ranking of carbohydrates (e.g., Table 1), from 1 to 100, based on their ability/effect to raise blood glucose levels (Brand‐Miller and Buyken 2020). It provides an estimation of how quickly the carbohydrates in a food are digested and absorbed into the bloodstream (Vlachos et al. 2020). Foods with GI > 70 are considered high GI foods and result in a quick rise in blood sugar levels when digested and metabolized whereas foods with GI < 55 produce a smaller and slower rise in blood sugar levels (Atkinson et al. 2021). Glycemic load is the product of both the GI and total amount of food consumed (Vlachos et al. 2020).

Knowledge of the GI of DP 1 to 3 saccharides is important as different compositions of these sugars in food can change the overall GI of the food. For example, honey with 28% fructose has a GI of 72 whereas 34% fructose honey has a GI of 48 (GI Database 2012). There are also calorie‐free sugars like l‐arabinose, saccharides that are not absorbed by the body nor metabolized and can act to inhibit the intestinal enzyme sucrase. In this way, l‐arabinose can prevent a rise in blood glucose caused by sucrose consumption (Seri et al. 1996; Wang et al. 2021).

### 1.4 What Function Do Saccharides Play in Food?

The degree a food is processed, such as drying (Fu et al. 2021), has a direct impact on its saccharide content (Wang et al. 2021), with changes noted in digestibility and physiological aspects (Zhang, Zhu, et al. 2022). Manufacturing processes based around either lactic acid or ethanol fermentation cause the largest changes to saccharide content. Lactic acid fermentation is a common manufacturing process for dairy products which affects the lactose, galactose and glucose content. As a result, products with long fermentation times, such as cheese, contain small amounts of saccharides (Monti et al. 2017). Ethanolic fermentation has a well‐known relationship to the starting ingredient's saccharide content. In products such as wine, saccharide content can also affect the final products' density, viscosity, astringency, oiliness, storage time, classification (sweet or dry), and pungency (Fa et al. 2017).

“Cooking” describes a range of processes that also impact on the saccharide content of foods and can occur in home kitchens, restaurants and commercial food manufacturing sites. Reducing saccharides in foods take part in the Maillard reaction, in which the amino acids and saccharides react, resulting in a darker appearance and a range of flavors and aromas. These Maillard reaction products become precursors for several groups of aroma compounds including aldehydes, ketones, alcohols, sulfur compounds, acids, and nitrogen heterocyclic compounds (Ge et al. 2021; Liu et al. 2024). Additionally, saccharides can also act in tandem with these and other aroma compounds to provide a richer sensory experience to the consumer (Fu et al. 2021).

Another reaction primarily involving saccharides is caramelization which occurs during baking and candy‐making (Pico et al. 2015). Aroma compounds formed by monosaccharides caramelizing include oxygen heterocyclic compounds such as furans (Liu et al. 2024). A further effect observed in cooking, is the *Lobry de Bruyn–Alberda van Ekenstein* transformation, best known as the process that converts < 25% total lactose to lactulose (which isn't absorbed by the body) when milk is heated (Nagasawa et al. 2017). This only occurs to a small percentage of the total saccharides and converts some of the aldose to the corresponding ketose (Nagasawa et al. 2017) in an equilibrium process. It is thus apparent that being able to quantify saccharides accurately in food addresses both public health and manufacturing quality issues. It can also have direct economic consequences, as returns to growers in industries producing fruit juice (Zhang and Ritenou 2016) and sugar cane, are tied to these results.

## 2 How Are Saccharides Currently Analyzed?

Despite the large number of analytical methods published for the quantification of saccharides in food, no “gold standard” exists that is widely accepted and free from limitations (Meyer et al. 2022). The choice of method depends on the sample type as well as the analytical objectives (Meyer et al. 2022). All sugars in food are analyzed by a two or three step process comprising of extraction, derivatization and quantification (instrumental analysis). The analytical technique used dictates whether derivatization is needed (or partially needed) depending on the analysis required.

### 2.1 Extraction

Foods contain a diverse array of substances such as water, lipids, proteins, vitamins, minerals, carboxylic acids, and polyphenols, all of which potentially interfere with saccharide determination. Separating target saccharides from these substances is desirable for accurate quantification and typically consists of extraction and filtration (Sanz and Martínez‐Castro 2007).

Sample extraction involves the removal of the analyte from its matrix. As saccharides are readily water soluble, this is usually completed by adding either water or a buffered solution to the sample matrix. For liquid matrixes this can involve dilution while for solid matrixes this involves blending or maceration with an aqueous solution. In matrixes with high protein content, Carrez clarification solutions are usually added to help precipitate protein (Muntean 2022). In samples high in fat, defatting by ether extraction may also be a prerequisite.

Filtration may be necessary to remove insoluble substances that can clog analytical columns (Kurzyna‐Szklarek et al. 2022). In some cases, this is replaced by centrifugation, as the undesired larger molecules can be precipitated out of solution using centrifugal force. Additionally, solid phase extraction (SPE) columns such as C18, porous graphitic carbon, aminopropyl silica, or ion exchange media can be used to remove other interfering soluble organic or inorganic compounds (Kurzyna‐Szklarek et al. 2022).

#### 2.1.1 Extraction for Free Sugars Analysis

Saccharides in food are present as either free (and therefore easily extractable) or bound in larger, more complex compounds (Cooper 2022). Free sugars are easier to analyze and can usually be successfully extracted by dilution and filtering. Matrix interference is observed in samples with high levels of fat, protein, or complex carbohydrates and requires their removal or reduction for the accurate quantitation of free saccharides.

#### 2.1.2 Hydrolysis for Bound Sugars Analysis

Saccharides bound in polysaccharides, oligosaccharides or other compounds containing sugar moieties can be broken down via hydrolysis to their constituent monomers. An application of this process is the hydrolysis of starch to make sweeteners such as high fructose corn syrup (HFCS), commonly used by the baking and brewing industries (Pico et al. 2015). Hydrolysis of bound sugars, prior to analysis, is either acid‐ and/or enzyme‐based and is a balance between achieving maximum depolymerization with minimal degradation of the liberated monosaccharides (Liu et al. 2021). Acid hydrolysis is the most widely used method and typically employs sulfuric acid (H_2_SO_4_) catalysis, although trifluoracetic acid (TFA) and hydrochloric acid (HCl) have also been used (Kurzyna‐Szklarek et al. 2022). Factors to consider for acid hydrolysis are: acid types, concentration, temperature, and treatment time (Liu et al. 2021). The removal of the remaining acid, after the reaction is complete, should also be carefully considered since some processes may affect the resulting sugar monomers and introduce impurities and artifacts. The advantages of acid hydrolysis are that it is (a) less time consuming and (b) more cost‐effective than other hydrolysis methods (Suksom et al. 2015). Another advantage of using standardized hydrolysis methods is that results are easily comparable since it is regularly used in the food industry.

Enzymatic hydrolysis is another popular method of hydrolyzing bound sugars with minimal degradation of the liberated monosaccharides (Liu et al. 2021). Described as a gentler and more specific method, it uses enzymes such as β‐glucosidase, α‐amylase, pectinase, cellulase, or xylanase (Liu et al. 2021). Factors that influence enzyme hydrolysis include the choice of enzyme, temperature, pH, enzyme concentration, activity and stability. After hydrolysis, enzymes need to be deactivated, usually by placing samples in a boiling water bath. Complex polysaccharides may need more than one enzyme for full hydrolysis (Liu et al. 2021) due to the specific action of the enzymes. Important factors to consider for enzymatic hydrolysis include cost of enzymes, suitable storage, effective lifespan, and requirements for any subsequent derivatization.

### 2.2 Derivatization

The degree of derivatization depends upon the target analyte, sample type, separation process, and the analytical instrument to be used. As a rule of thumb, derivatization should be kept to a minimum due to (a) the time‐consuming and complex process involved (Liu et al. 2021), (b) the use of noxious or toxic chemicals (Ellingson et al. 2016), and (c) the potential introduction of unwanted by‐products and artifacts into the sample (de Souza et al. 2019; Vennard et al. 2020). Derivatization prior to high performance liquid chromatography (HPLC) or capillary electrophoresis (CE) may be necessary to introduce chromophores, fluorophores or electrical charge to the analyte to facilitate separation and/or detection. The most common derivatizing agent for both HPLC and CE is 1‐phenyl‐3‐methyl‐5‐pyrazolone (PMP) (Acunha et al. 2016; Meyer et al. 2022), which also creates several contaminant reagent peaks (Liu et al. 2021). Other derivatizing agents include 8‐aminopyrene‐1,3,6‐trisulfonate, 2‐aminopyridine, 8‐aminonaphthalene‐1,3,6‐trisulfonate, and 6‐aminoquinoline. Typically, fluorophores are added by a process of reductive amination, a process that can be difficult to reproduce. In addition, some sugars (such as fructose) are not easily amenable to derivatization (Liu et al. 2021) and non‐reducing saccharides (e.g., sucrose) cannot undergo reductive amination.

Due to the thermal instability and low volatility of saccharides, derivatization is a requirement prior to analysis by gas chromatography (GC). Different approaches for this have been created (Meyer et al. 2022) with acetylation, silylation and methylation being common (Liu et al. 2021). Each method has specific limitations (Kurzyna‐Szklarek et al. 2022), such as co‐eluting peaks (Liu et al. 2021) usually caused by silylation of multiple anomeric forms of a sugar giving complex chromatograms. It is worth noting that some analytical techniques such as ion chromatography (IC) do not require derivatization, which is a major advantage over other techniques.

## 3 Quantification (Instrumental Analysis)

Methods for total sugar analysis have existed since the 17th century and include physical methods such as refractometry (measurement of the sample solution's ability to bend light), hydrometry (measurement of the density of a solution), and polarimetry (measurement of the solution's ability to rotate polarized light). These methods are limited by a lack of specificity and can respond to other non‐saccharide dissolved substances within the sample solution (Bates 1942). Chemical methods, such as copper sulphate (Benedict's or brick test) and silver nitrate (Tollen's or mirror test) bring a degree of specificity, as these typically react with reducing saccharides to form a metallic precipitate (Pratt and Cornely 2014). While these are more specific than physical detection methods, specificity is still lacking with these chemical methods since other reducing substances can also react, while creating large amounts of heavy metal waste.

The most common saccharide analysis technique used in food products is enzymatic analysis, which relies on the reaction of specific enzymes to quantify saccharides (mainly glucose and fructose) (Al‐Mhanna et al. 2018). Enzymatic reactions are monitored via changes in color, voltage or pH. There is no standardized method for this analysis across all saccharides, with several companies providing kits with their own tailored methods. These analyses are cost‐effective (Tzouwara‐Karayanni and Crouch 1990) and accessible to laboratories that lack chromatographic instruments. However, they have major drawbacks such as (a) available enzymes are specific to a certain saccharide only, (b) enzymes can potentially react with other compounds in the sample, and (c) may not be suitable for novel food samples with unknown sugar compositions (Al‐Mhanna et al. 2018).

Apart from enzymatic analysis, most analysis of saccharides is performed using one of four main chromatographic techniques: HPLC, GC, CE, or IC coupled with a variety of detectors. Each technique and detector combination has advantages and disadvantages dependent upon matrix complexity, amount of information required, cost, and complexity of analysis (Vennard et al. 2020). As saccharides do not have native chromophores or fluorophores, analysis using either visible light absorbance or fluorescence detectors requires derivatization.

### 3.1 High Performance Liquid Chromatography (HPLC)

**FIGURE 2:**
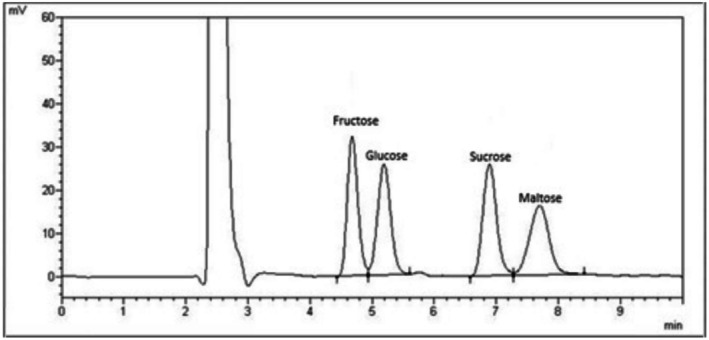
Typical chromatogram of four saccharides by HPLC/RID.

As saccharides are readily soluble and HPLC instruments are a common presence in analytical labs, HPLC is the most widely used instrumental techniques for saccharide analysis. Of the detectors available, HPLC coupled with either refractive index detection (RID) or evaporative light scattering detector (ELSD) are most common. RID relies on the saccharides ability to refract light in solution, whereas ELSD operates on the ability of the analyte to scatter light after nebulization. Both detectors are non‐specific for saccharides since most compounds also refract or scatter light and will create an indistinguishable response. An example of the resulting RID generated chromatograph can be seen in Figure 2. Interferences from complex matrixes are frequently observed and it is recommended that HPLC/RID should only be used with simple food sample types (Vennard et al. 2020).

RID response is also sensitive to changes in temperature, pressure, and solvent composition (Monti et al. 2017), hence, isocratic elution is the only practical mode of quantitative analysis. This limitation makes method optimization in RID more complicated since complex samples might require longer run times to achieve good separation without gradient ramps to facilitate faster elution. An example is the analysis of DP 3–5 oligosaccharides in chickpea meal matrix by Gangola et al. (2014) who reported analysis times of 190 min (160 min run time + 30 min wash) for good separation of raffinose (DP 3), stachyose (DP 4), verbascose (DP 5) along with the DP1, and DP2 saccharides present. The 160 min run time was a result of using a 0.1 mL/min flow rate to circumvent the high back‐pressure experienced during the runs while the 30 min wash was performed to increase the number of samples that can be injected to ~50 samples before replacing the guard column.

ELSD offers better baseline stability than RID and is generally unaffected by changes in flow rate, temperature (Magwaza and Opara 2015), or solvent composition (Liu et al. 2021). For these reasons, ELSD is used more often for saccharides analysis than RID (Kurzyna‐Szklarek et al. 2022). A limitation of ELSD is the need for a nebulizer gas, as well as the “correct ratio” of nebulizer gas to the composition of the mobile phase and concentration of the analyte. In addition, the analytes need to be non‐volatile compared to the mobile phase. A positive correlation is reported with respect to the detector response to the concentration of organic solvent in the mobile phase (Kurzyna‐Szklarek et al. 2022). Therefore, as the amount of solvent is increased to promote volatility needed for analysis, the level of detection decreases. Another well‐known limitation is the non‐linear (sigmoidal or exponential) response observed, (Kurzyna‐Szklarek et al. 2022) which requires either polynomial calibration curves or use of a bi‐logarithmic scale (log of both axes) post‐analysis (Magwaza and Opara 2015).

Both RID and ELSD are not suitable for trace saccharide analysis (< 1 mg/L concentrations). Since mono‐ and disaccharides are typically found in large quantities in both processed and unprocessed food, this is not a limitation for routine food analysis. The continued use of these detection methods is mainly due to economic reasons, as both detectors are cheaper than other options. Both detection methods also do not involve derivatization as is needed for other analysis such as GC.

### 3.2 Gas Chromatography (GC)

GC has over 60 years of history in saccharide analysis (Liu et al. 2021). It is reported to have excellent sensitivity (Vennard et al. 2020) and separation (Kurzyna‐Szklarek et al. 2022) on par with HPLC, while still being a simple instrument to use (Liu et al. 2021). GC is often paired with a flame ionization detector (FID) for saccharides analysis which measures the conductivity of a small flame as the eluent is burnt. Since FID is a nonspecific detector, it has been replaced in favor of more specific detectors such as a mass spectrometer.

**FIGURE 3:**
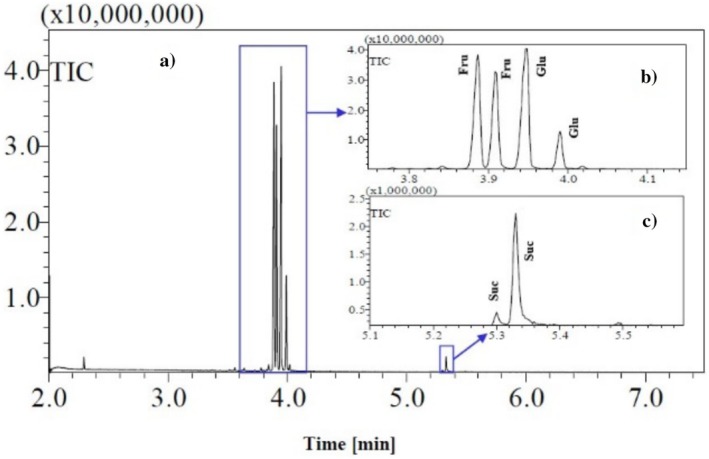
Example GC/MS chromatogram of oximated‐silylated date juice sample, with two peaks observed for each sugar. (A) Whole chromatogram, (B) Zoom‐in of peaks for fructose (Fru) and glucose (Glu). (C) Zoom‐in of peaks for sucrose (Suc) (adapted from Al‐Mhanna et al. (2018) with permission).

The main downside to saccharide analysis by GC is the derivatization required for analysis. Liu et al. (2021) reported that each derivatization method is only effective with certain groups of saccharides and multiple derivatization procedures may be required when multiple groups of saccharides are present. A problem unique to GC‐based analysis is that derivatized saccharides are exceedingly volatile at low temperatures, with full analyte recovery requiring expertise and due diligence. A well‐known drawback is that certain derivatization reactions can create multiple products from a single saccharide (Figure 3) (Al‐Mhanna et al. 2018; Vennard et al. 2020) which results in additional time needed for post‐analysis data processing (Kurzyna‐Szklarek et al. 2022). For these reasons, GC analysis is reported to be less cost‐effective than HPLC (Sadeghi et al. 2022).

### 3.3 Capillary Electrophoresis (CE)

While not as common as HPLC or GC for saccharide analysis in food, CE is a versatile analytical technique reported to have high separation efficiency and short separation times (Acunha et al. 2016). CE only requires small amounts of sample and reagents which also leads to low waste production. The technique can be paired with a variety of optical (photo diode array and fluorescence detection after derivatization), electrochemical (conductivity and electrochemical), and MS detectors (Kurzyna‐Szklarek et al. 2022). As these detectors are mostly the same as those used for HPLC, it has been suggested as a replacement for HPLC in several applications as sample processing or elution times are typically faster.

Separation by CE depends on the charge of the analyte (Kurzyna‐Szklarek et al. 2022). As most natural saccharides are not readily ionized, analysis by this technique has been limited (Mantovani et al. 2018). Saccharides are also “slow eluting” when compared to the generically quick paced CE runtimes, with some methods taking as long as the equivalent HPLC methods they were reported to replace (Kurzyna‐Szklarek et al. 2022). Over time, improvements have been made to CE methods, making them more suitable for both derivatized and underivatized saccharide analysis (Mantovani et al. 2018). The most common option is the use of pre‐column derivatization, a time and cost‐intensive process that provides lower limits of detection but has similar elution times to HPLC (Magwaza and Opara 2015). Another option is to ionize the saccharides in a high pH solution and allow the resulting ions to complex with borate or copper. This method can lead to poor sensitivity and selectivity due to side reactions with other compounds that might be present (Mantovani et al. 2018). Analysis without complexation at high pH is also possible (Mantovani et al. 2018) but requires a suitable electrochemical detector which is not widely available for CE (Liu et al. 2021).

A recent study by Kurzyna‐Szklarek et al. (2022) has reported that CE is not suited for identifying uncommon saccharides in foods due to ambiguity between saccharides and organic acids. The potential reaction of sodium dodecyl sulphate (SDS), a common mobile phase component in CE, with proteins and lipids present in food samples is also a reported drawback of CE (Mantovani et al. 2018). Despite these drawbacks, simple methods for the analysis of glucose, fructose and sucrose in juice (Zhang and Ritenou 2016), maltose and glucose in beer, as well as maltose, lactose and glucose in milk have been reported (Acunha et al. 2016). These studies cover only the major saccharides for each sample type, demonstrating limitations with the instrumentation compared to the other instruments presented.

### 3.4 Ion Chromatography Coupled With Pulsed Amperometric Detection (IC/PAD)

**FIGURE 4:**
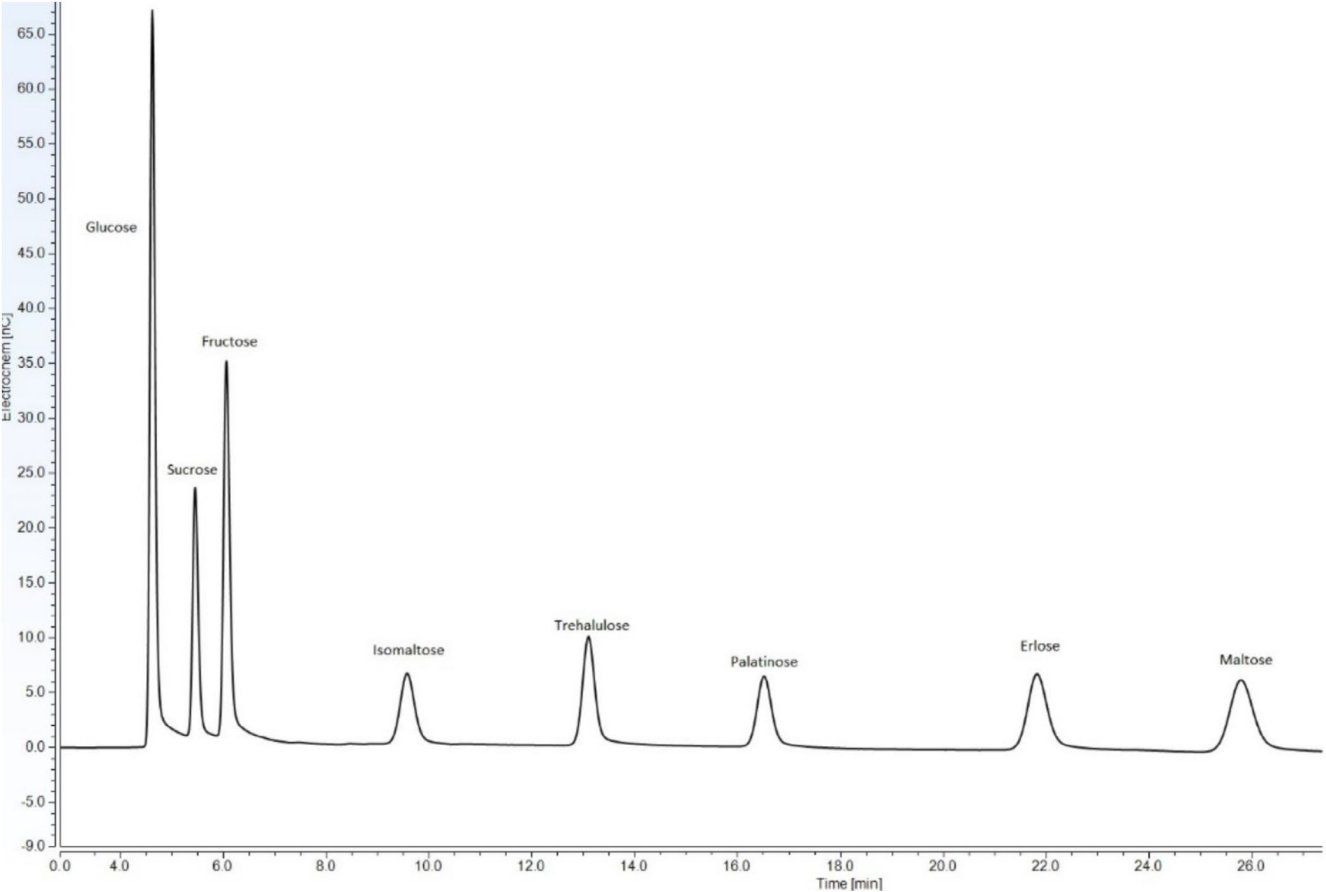
Example chromatogram of the analysis of eight saccharides by IC/PAD.

Analysis of saccharides by IC operates on the principle that saccharides are weak acids. When dissolved in a mobile phase of high pH, the molecules lose acidic hydrogen to form anions which interact with the column and typically elute in the order of increasing ionic strength; mono‐, di‐, then oligosaccharides (Figure 4) (Anders et al. 2015). Common mobile phases include aqueous sodium or potassium hydroxide, but sodium acetate has also been used to accelerate the elution of strongly bound species without compromising selectivity or interfering with detection. Acetate interacts more strongly with anion exchange sites, increasing the mobile phase solubility for oligo‐ and polysaccharides (Eggleston et al. 2016). IC is commonly paired with pulsed amperometric detection (PAD) for the analysis of saccharides as this detector is compatible with the highly caustic mobile phase (Kurzyna‐Szklarek et al. 2022).

Saccharide analysis by IC/PAD, also known as high performance anion exchange chromatography/pulsed amperometric detection (HPAEC/PAD) (Eggleston et al. 2016), was first demonstrated by Hardy et al. in 1988 (Gudlavalleti et al. 2014; Hardy et al. 1988; Liu et al. 2021). Stationary phases can be customized for specific saccharides to provide great selectivity and are increasingly used in the analysis of food (Malacarne et al. 2016; Vennard et al. 2020). IC/PAD methods are able to analyze saccharides up to DP 80 (Eggleston et al. 2016) with picomole level sensitivity (≈180 pg/L) (Zhang, Zhu, et al. 2022).

Similar to RID and ELSD, PAD does not require molecules to be derivatized (Liu et al. 2021). PAD can detect both reducing and non‐reducing sugars due to the oxidation of hydroxyl and aldehyde groups caused by the potentiometric waveform generated by a noble metal electrode (Kurzyna‐Szklarek et al. 2022). IC/PAD has also been used to analyze food for flavonoids, lignans, amino acids (Perini et al. 2018), organic acids, sugar alcohols, and acids (de Souza et al. 2019).

#### 3.4.1 IC/PAD Analysis Advantages

IC/PAD has several advantages over other chromatographic instrumentation, including:

- No derivatization is needed. This makes analysis cost‐efficient as the usual reagents, equipment, and waste disposal (including organic solvents (Zhang, Zhu, et al. 2022)) required for derivatization are not needed. The lack of noxious or toxic chemicals (Ellingson et al. 2016) also provides positive safety and environment‐friendly credentials to this instrumentation.
- Minimal sample preparation is required. Due to IC/PAD having high saccharide analyte sensitivity, minimal amounts of sample are needed for analysis (Liu et al. 2021) and minimal cleanup is required (Vennard et al. 2020). Common interfering or competing matrix compounds are considerably diluted and do not cause the same problems encountered with other chromatographic instrumentation. As such, the sample preparation for ion chromatography is affectionately referred to as “dilute and shoot.”
- Detector sensitivity. PAD is not sensitive to changes in salt concentration (ubiquitous in food) (Magwaza and Opara 2015) and has a response that is linear over three orders of magnitude, allowing for the simultaneous determination of a number of naturally occurring compounds (Malacarne et al. 2016). Unlike other detectors, PAD operates by direct quantitation, as the saccharides must interact with the detector to generate a signal (Visvanathan et al. 2021). It is this direct interaction that affords limits of detection orders of magnitude below HPLC/RID and GC/MS (Anders et al. 2015). Due to this, IC/PAD is currently being used for the routine quantitative analysis of saccharides in a number of food industries (Zhang and Ritenou 2016).
- High resolution. Well‐known advantages of IC/PAD are its high resolution, selectivity (baseline separation), and sensitivity (Liu et al. 2021; Magwaza and Opara 2015). There are minimal issues with differentiating between groups of saccharides with several studies showing multiple mono‐, di‐, and oligosaccharides being effectively separated in a single run (Ellingson et al. 2016).
- Direct observation of components in saccharide hydrolysis reactions. Typically, only dilution and filtration are required for hydrolysis product analysis by IC/PAD. Other instruments require derivatization or other reactionary chemical processes, in order for products of hydrolysis to be observed. These additional processes can result in further reactions taking place which can cause components to be undetected, converted into interfering compounds, or be misidentified. Only the IC/PAD has the capability to do this when not paired to a mass spectrometer (Visvanathan et al. 2021).

#### 3.4.2 IC/PAD Analysis Disadvantages

IC/PAD does have several disadvantages, including:

- Non‐specificity of technique. One of the main drawbacks of IC/PAD is that it is a non‐specific technique that lacks any secondary characterization (or confirmation) of analytes. Co‐elution has been reported for mono‐, di‐, and amino‐saccharides, sugar alcohols (Vennard et al. 2020), vitamins, amino acids and proteins (Ni et al. 2016).
- Specialized equipment, maintenance, and considerations. Often a purpose‐made instrument is required for analysis, as Liu et al. (2021) reports the high pH mobile phase used in IC can have an undesired effect (damage) on routine HPLC equipment. Because of the high pH of the mobile phase used in IC, it requires the use of specific tubing and parts which are different to what regular HPLC equipment utilizes. Therefore, switching to an IC‐based methodology is not as simple as switching columns and mobile phase, as customary to HPLC practices. Choice of mobile phase should be carefully considered as carbonate created from the reaction of hydroxide in the eluent with atmospheric carbon dioxide results in an undesirable acceleration of analyte elution. This is also necessary for the maintenance of the detector, which needs hours to equilibrate before starting analysis (Ellingson et al. 2016).
- Possibility of co‐elution for certain saccharides. Even with the use of gradient elution to separate different ionizing saccharide groups (Nouara et al. 2019), certain pairs of monosaccharides such as rhamnose and arabinose (Liu et al. 2021) and xylose and mannose have been reported to elute close to each other (Magwaza and Opara 2015).
- Long analysis times. Lengthy sample analysis times (ca. 90 min) have been reported (Fels and Bunzel 2022).

### 3.5 Complementary Mass Spectrometry

One way to overcome the limitations of non‐specific detectors is the addition of a mass spectrometer to any of the separation techniques (HPLC, GC, CE, or IC) as the sole or additional detector (Kurzyna‐Szklarek et al. 2022). The use of a mass spectrometer as a single “standalone universal detector” for HPLC and GC has increased in recent years (Meyer et al. 2022). Many methods have been developed over the last few years to utilize the sensitivity, specificity, simplicity and shorter run times a MS affords (Kurzyna‐Szklarek et al. 2022).

Unfortunately, saccharides are not well suited for MS analysis due to their hydrophilic, neutral nature which results in inefficient desolvation and poor ionization when using electrospray ionization (ESI) (Kurzyna‐Szklarek et al. 2022; Mantovani et al. 2018). One approach to counter this problem is to generate an adduct with inorganic ions including PO_4_^3−^, NO_3_^−^, or SO_4_^2−^ in negative ion mode or Li^+^, Na^+^, K^+^, Cs^+^, or NH_4_^+^ in positive ion mode (Kurzyna‐Szklarek et al. 2022). This approach has drawbacks as involatile buffers can interfere with MS performance and ion generation (Sargent 2013). Another approach is pre‐column derivatization with aromatic reductive amination reagents, such as 2‐aminoacridone or 2‐aminopyridine, although loss of sensitivity towards some polysaccharides has been reported (Mantovani et al. 2018). Atmospheric pressure chemical ionization (APCI) is not commonly used since the weak acidic character of saccharides results in low ionization efficiency in negative ion mode (Kurzyna‐Szklarek et al. 2022). There have been limited reports on the use of positive ion mode APCI for saccharide analysis.

The main limitation of MS detection is that most saccharides are isomers of each other, that is, they have the same molecular mass (see for example Table 1). Therefore, unless the separation technique provides a high degree of selectivity, co‐eluting saccharides may not always be further resolved by the MS (Jeong et al. 2009), although other potential co‐eluents can be distinguished. In some laboratories, especially food production laboratories requiring low‐cost analysis, a MS is prohibitively expensive for the analysis of percentage level saccharides (g/L or g/kg) (Ni et al. 2016; Pico et al. 2015).

## 4 Saccharide Analysis of Food by IC/PAD in the Past Decade

### 4.1 Analysis of Free Saccharides in Foods by IC/PAD

As a demonstration of the resolving power of the IC/PAD, Mudrić et al. (2017) stated that only glucose, fructose, and sucrose were previously reported in paprika but using IC/PAD they were able to report the presence of arabinose, ribose, rhamnose, xylose, mannose, trehalose, maltose, and raffinose. Wang et al. (2021) found more saccharides in jujubes than previously reported by GC. Akšić, Tosti, et al. (2019) reported finding more saccharides than previously reported in strawberries and blueberries, which provided a means to differentiate between organically and non‐organically grown berries.

Gangola et al. (2014) compared IC/PAD and HPLC/RID methods for the analysis of chickpea saccharides and reported having high back pressure problems with HPLC/RID which were only resolved by replacing the guard column after 50 samples. Additionally, HPLC/RID required longer run times (ca. 190 min) for raffinose, stachyose and verbascose, compared to IC/PAD, to achieve good separation between these saccharides and the DP1 and DP2 saccharides present. The sensitivity of IC/PAD was evident in multiple studies involving saccharides in cheese and dairy products by (Monti et al. 2017; Monti et al. 2019). These studies could detect concentrations of lactose in Gorgonzola 100 times lower than required by the Italian government for “lactose‐free” labelling (Monti et al. 2019).

Several authors reported that the use of SPE for sample preparation prior to IC/PAD, especially for complex sample types, was effective and improved detection of saccharides. A study by Hu et al. (2017) reported a time‐consuming method for extraction of soluble rice saccharides requiring repeated 50% aqueous ethanol extractions. In contrast, Nešović et al. (2021) used C18‐E SPE to remove polyphenols and pollen to analyze 14 saccharides in buckwheat while Ispiryan et al. (2019) used polyamide SPE for a variety of cereals to analyze eight saccharides, with neither study encountering issues similar to Hu et al. (2017). The same observations about SPE are echoed with wine, well known for its high content of polyphenols, several of which co‐elute with monosaccharides. Fa et al. (2017) used polystyrene‐divinyl benzene SPE to remove polyphenols and resveratrol for a method that simultaneously analyzed seven saccharides and most amino acids. In contrast, de Souza et al. (2019) reported that without prior SPE cleanup, their method could only analyze three saccharides as well as a limited number of organic acids and sugar alcohols in wine.

Some sample types in Table 2, such as fruits, vegetables, and juices did not require SPE for sample preparation, instead dilution and centrifugation were sufficient. Wang et al. (2021) analyzed various fruits and vegetables and found a non‐linear maltose response due to an interfering peak of sodium acetate from the mobile phase. In contrast, Zhang, Zhu, et al. (2022) and Xiao et al. (2022) studied sweet corn and durian respectively, and both reported maltose without noting such an issue. Sadeghi et al. (2022) demonstrated the resolving power of the IC/PAD by detecting low concentrations of galactose alongside the major saccharides glucose and fructose present in potatoes.

**TABLE 2:** Summary of sugar analysis in various food sample types by IC/PAD during the last 10 years.

Samples	Analytes	Sample pretreatment	Chromatographic conditions	Calibration range (min–max)	References
Balsamic vinegar	Ara, Fru, Gal, Glc, Mal, Man, Rha, Suc, Xyl	H_2_O	Instrument: ICS 5000 Column: PA200 Elution: ND RE: Pd/PdH		(Perini et al. 2018)
Cereal, buckwheat	Ara, Fru, Gen, Glc, Iml, Mal, Mal3, Mel, Pan, Raf, Suc, Tre, Tur, Xyl	H_2_O Centrifugation C18‐E SPE	instrument: ICS 3000 column: PA100 elution: ND	0.9–100 μg/L	(Nešović et al. 2021)
Various cereals	Fru, Gal, Glu, 1ke, Lac, Mel, Raf, Suc	H_2_O MeOH and NaN_3_ Carrez solution Centrifugation Polyamide SPE	Instrument: ICS 5000 Column: PA100 for mono and di Column: PA200 for oligo and poly Elution: ND RE: Pd/PdH	0.1–1 mg/L and 1–20 mg/L	(Ispiryan et al. 2019) (Ispiryan et al. 2020)
Rice	Fru, Glc, Mal, Suc, Raf	50% EtOH Centrifugation	Instrument: 850 professional Column: Carb 1 Elution: Isocratic RE: Pd/PdO Waveform 1 ^a^	1–10 mg/L	(Hu et al. 2017)
Chickpeas	Glc, Fru, Raf	80% EtOH 60°C incubation for 45 min Centrifugation C18 SPE	Instrument: ICS 5000 Column: PA100 Elution: gradient ^a^ Waveform 2 ^a^	6.2–100 mg/L	(Gangola et al. 2014)
Various dairy products	Fru, Gal, Glc, Lac, Mal, Suc	Various	AOAC trial data column: PA1 Instrument: ICS 5000 Column: PA20 Column: PA200 for lactose and maltose Elution: gradient^a^		(Brunt et al. 2021)
	All, Fru, Gal, Glc, Lac, Mal, Suc	Carrez solution centrifugation		1–10 mg/L	(Fels and Bunzel 2022)
	Ara, Fru, Fuc, Gal, Glc, Lac, lactulose, Mal, Mal3, Mel, Pal, Suc, Tre	Centrifugation EtOH Carrez solution Centrifugation	Instrument: ICS 3000 Column: PA1 Elution: gradient^a^		(Sanders et al. 2019)
Cheese	Gal, Glc, Lac	Boiling water with Ultra Turrax Centrifugation Carrez solution SPE	Instrument: Ultimate 3000 with ECD 3000 RS ED Column: PA20 Elution: isocratic RE: Pd/PdH	0.3–5 mg/L	(Monti et al. 2017) (Monti et al. 2019)
Fruit (blueberry & strawberry)	Ara, Fru, Gal, Rib, Glc, Iml3, Mal, Mal3, Suc, Tre, Tur, Pan, Xyl, Mel, Rha, Raf, Iml	H_2_O	Instrument: ICS 3000 Column: PA100 Elution: gradient ^a^	0.9–100 μg/L	(Akšić, Tosti, et al. 2019)
Fruit and veg	Ara, Fru, Gal, Glc, Mal, Suc	H_2_O Centrifugation	Instrument: ICS 3000 Column: PA20 Elution: gradient ^a^ Waveform 5 ^a^	0.5–15 mg/L	(Wang et al. 2021)
Durian	Fru, Glu, Mal, Rib, Suc	80% EtOH Centrifugation	Instrument: ICS 5000 Column: PA1 Elution: gradient ^a^	0.5–50 mg/L	(Xiao et al. 2022)
Tropical fruit	Fru, Glc, Suc		Instrument: 861 advanced Column: Carb 1 Elution: isocratic	5–80 mg/L	(L. Yu et al. 2016)
Chinese date	Fru, Glc, Suc	H_2_O Centrifugation	Instrument: ICS 3000 Column: PA 20 Elution: gradient ^a^		(Fu et al. 2021)
Honey (common sage)	Fru, Glu, IMl, IMl3, Mal, Mal3, Suc, Tre, Tur	H_2_O Filtration	Instrument: ICS 3000 Column: PA 10 Elution: gradient ^a^	1–10 mg/L	(Gašić et al. 2015)
Honey (manuka)	Erl, Fru, Glc, Koj, Mal, Mal3, nigerose, Pan	10% EtOH with charcoal filter 50% EtOH EtOH evaporation	Instrument: ICS 3000 Column: PA 100 Elution: gradient ^a^		(Lane et al. 2019)
Honeydew honey	Erl, Fru, Glc, Lac, Mal, Raf, Rha, Suc	Column switching cleanup	Instrument: ICS 3000 Column: PA 10 Waveform 2 Elution: gradient ^a^	0.1–20 mg/L	(Ni et al. 2016)
	Erl, Fru, Glu, 1ke, Iml, Iml3, Pal, Mal, maltulose, Mel, Mlz, Suc, Tre	Centrifugation	Instrument: ESA HPLC Column: PA 10 Elution: isocratic	0.6–90 mg/L 1–80 mg/L	(Shaaban et al. 2020) (Shaaban et al. 2021)
Stingless bee honey	Erl, Fru, Glc, Mal, Suc, trehalulose	H_2_O Filtration	Instrument: Integrion Column: PA 210 Elution: isocratic Waveform 2 ^a^	1–50 mg/L	(Hungerford et al. 2021) (Zhang, Hungerford, et al. 2022)
Infant formula	Fru, Gal, Glc, Lac, Suc		Instrument: ICS 5000 Column: PA 1 Elution: gradient ^a^		(Zhang et al. 2016)
Juice, citrus	Fru, Glc, Mal, Suc	Centrifugation AEC	Instrument: ICS 3000 Column: PA 200 Elution: isocratic		(Zhang and Ritenou 2016)
Millet beer mash	Fru, Glc, Mal, Mal3, Rha, Suc	Centrifugation	Instrument: ICS 5000+ Column: PA 20 with aminoTrap guard column Elution: gradient ^a^		(Ledley et al. 2023)
Spice, paprika	Ara, Fru, Glc, Mal, Man, Raf, Rha, Rib, Suc, Tre, Xyl	80% EtOH Centrifugation EtOH evaporation	Instrument: ICS 3000 Column: PA 10 Elution: gradient ^a^		(Mudrić et al. 2017)
Sweet sorghum syrup	Iml, Iml3, leucrose, Mal, Mal3, Pan		Instrument: Dionex BioLC Column: PA 1 Elution: ND Waveform 2 ^a^		(Eggleston et al. 2016)
Wine	Ara, Fru, Glc		Instrument: 850 professional Post‐column pump for PAD Column: PA 1 Elution: gradient ^a^ RE: Pd/PdO waveform 3 ^a^	2–30 mg/L	(de Souza et al. 2019)
	Ara, Fru, Gal, Glc, Man, Rib, Tre	PS‐DVB SPE 10% EtOH	Instrument: ICS 3000 Column: PA 10 Elution: gradient ^a^ Waveform 2 ^a^	0.1–10 mg/L	(Fa et al. 2017)
Vegetable, sweet corn	Fru, Glc, Mal, Suc	50% EtOH at 85°C for 1 h Centrifugation	Instrument: ICS 5000+ Column: PA 200 Elution: isocratic		(Zhang, Zhu, et al. 2022)
Potato	Fru, Gal, Glu	Juicer 70% EtOH Centrifugation	Instrument: ICS 6000 Column: PA 100	10–50 mg/L	(Sadeghi et al. 2022)

Abbreviations: 1Ks = 1‐ Kestose, All = allose, Ara = arabinose, Erl = erlose, Fru = fructose, Fuc = fucose, Gal = galactose, Gen = gentibiose, Glc = glucose, Iml = isomaltose, Iml3 = isomaltotriose, Koj = kojibiose, Lac = lactose, Mal = maltose, Mal3 = maltotriose, Man = mannose, Mel = melibiose, Mlz = melezitose, ND = not described, Pal = palatinose, Pd = Palladium, PdH = Palladium hydride, PdO = Palladium oxide, Pan = panose, Raf = raffinose, Rha = rhamnose, RE = Reference electrode, Rib = ribose, SPE = Solid phase extraction, Suc = sucrose, Tre = trehalose, Tur = turanose, Xyl = xylose.

^a^ Programs and waveforms used are listed in Supporting Information.

### 4.2 Honey Analysis by IC/PAD

Honey, often thought of as a simple matrix consisting mainly of water and saccharides, is defined as consisting of < 20% water, more than 60% reducing sugars, and only 5%–10% sucrose (Al‐Mhanna et al. 2018; Alimentarius 2022). What was not stated, is that honey also contains vitamins, amino acids, proteins and other organic compounds which can lead to damage to IC columns and erroneous results (Ni et al. 2016). It is characterized by the insect and nectar source from which it originated. In recent years, nectar and pollen have also been investigated as food sources for human consumption (Akšić, Gašić, et al. 2019). The major sugars in honey from bees of the *Apis* genus are glucose and fructose with arabinose, sucrose, trehalose, turanose, maltose and isomaltose reported as minor components (Gašić et al. 2015).

An emerging type of honey, popular in Central and Eastern Europe, is forest honey, also known as honeydew honey (Ni et al. 2016). This honey is a result of *Apis* bees collecting honeydew, a saccharide‐rich excrement of insects feeding on fir, pine, or chestnut trees (Shaaban et al. 2020) (Shaaban et al. 2021), during periods when floral nectar is scarce. Forest honey is reported to contain fewer monosaccharides, for example, glucose and fructose (Ni et al. 2016), and more di‐ and oligosaccharides, for example, trehalose, isomaltose, melezitose, raffinose (Lane et al. 2019), maltose and erlose when compared to nectar‐derived *Apis* honey (Shaaban et al. 2021). The presence of these additional di‐ and oligosaccharides were confirmed in forest honey by Shaaban et al. (2020) who found melibiose, isomaltose, turanose, maltulose, isomaltulose, maltotriose, isomaltotriose, and raffinose in all samples tested. Shaaban et al. (2021) also noted that there is no agreed international standard for forest honey.

Stingless bee honey is another honey for which no international standard currently exists. This honey originates from the *Meliponini* bees and is characterized by the presence of the novel saccharide trehalulose (Fletcher et al. 2020). IC/PAD was used by Hungerford et al. (2021) to analyze products of bee feeding experiments, with Zhang, Hungerford, et al. (2022) describing how the saccharide trehalulose was formed by the bees. Both these authors, along with Ni et al. (2016), used reverse phase pre‐treatment columns to remove co‐eluting organic compounds from honey prior to IC/PAD. In contrast to pre‐treatment columns, Lane et al. (2019) used loose activated charcoal, which was added directly to the sample solution to maximize surface reaction area. This charcoal reportedly absorbed only oligosaccharides of interest from the solution, leaving the co‐eluting monosaccharides. The oligosaccharides were then extracted from the charcoal. Ni et al. (2016) stated that the use of pre‐treatment columns was commonplace in honey analysis, whereas published studies that use these columns appear sporadic.

### 4.3 Oligosaccharide Elution Pattern Fingerprinting Using IC/PAD as a Guide for Authenticity

The use of analytical techniques to detect and protect against food fraud is an ongoing area of research. Typically, the fraud involves either dilution of a high value product with a low‐cost additive to extend the product or substitution of a lower quality product in place of a higher quality product. The resolving power of IC/PAD for free oligosaccharides has allowed for the authenticity “fingerprinting” of numerous manufactured products, including those from sugar crops (Eggleston et al. 2016). Due, however, to a lack of sufficient oligosaccharide reference materials, identification of components by retention times was not possible for all components (Lane et al. 2019).

IC‐based fingerprinting methods appear to have improved over time. Eggleston et al. (2016) used IC/PAD to determine if sweet sorghum syrups had been diluted with lower cost sweeteners but were not able to determine provenance. In the same year, Zhang and Ritenou (2016) used maltose as an indicator that citrus leaves had been used to bulk orange or grapefruit juices. Perini et al. (2018) was able to successfully demonstrate the provenance of balsamic vinegars by this technique, while also establishing maltose as a good indicator of adulteration. Hungerford et al. (2021) and Zhang, Hungerford, et al. (2022) suggested that the presence of trehalulose is an indicator of stingless bee honey authenticity. Lane et al. (2019) was also successful in using oligosaccharide fingerprinting by IC/PAD to determine Manuka *Apis* honey authenticity.

### 4.4 Bound Sugar Analysis by IC/PAD Following Hydrolysis

Similar to the literature for free saccharides, presented in Table 2, no single method of sample preparation or analysis is dominant for bound saccharides across all sample types in Table 3. To quantify the bound saccharides released by hydrolysis, the concentration of free saccharides in samples must first be determined and subtracted from what is found after hydrolysis. This process was completed before hydrolysis for all oligo‐ and/or polysaccharide samples reported by all authors.

**TABLE 3:** Summary of published methods on analysis of hydrolyzed sugars in various food sample types by IC/PAD during the last 10 years.

Samples	Analytes	Hydrolysis method	Chromatographic conditions^a^	Calibration range (min–max)	References
Cereal, wheat flour	Glc, Iml, Mal Mal3	Enzymatic hydrolysis α‐amylase from *A. oryzae* α‐amyloglucosidase from *A. niger*	Instrument: 850 professional IC Column: RCX‐30 Elution: gradient ^a^ RE: Pd/PdO Waveform 4 ^a^	2–13 mg/L	(Pico et al. 2015)
fruit (strawberry tree berries)	Ara, Fuc, Gal, Glc, Rha, Xyl	H_2_SO_4_ hydrolysis	Instrument: ICS 5000 Column: SA10 Elution: isocratic		(Marques et al. 2022)
Prebiotics	Gal, Glc, Lac	Enzymatic hydrolysis β‐galactosidase from *A. oryzae*	Instrument: ICS 5000 Column: PA 20 Elution: gradient ^a^	1–25 mg/L	(Lin et al. 2018)
Prebiotic, fruit derived	Ara, Fuc, Fru, Gal, Glc Rha, Rib, Xyl	H_2_SO_4_ hydrolysis	Instrument: ICS 5000 Column: PA 1 Elution: gradient ^a^ Waveform 2 ^a^		(Villacís‐Chiriboga et al. 2023)
Prebiotics	Ara, Fuc, Fru, Gal, Glc, Man, Rha, Xyl	Enzymatic hydrolysis β‐xylanase from *H. hemicellulosilytica* (x3) β‐xylanase from *C. stercorarium* (x1) Cellulase from *C. thermocellus* (x1) Endoglucanase from *C. thermocellus* (x1) Xylosidase from *H. hemicellulosilytica* (x1) Arabinofuranosidase from *C. stercorarium* (x1)	Instrument: ICS 3000 Column: PA 1 Elution: gradient ^a^ Waveform 2 ^a^	3–394 mg/L	(Mechelke et al. 2017)
Sugar, raw	Ara, Fru, Glc, Lac, Rib, Suc, Xyl	HCl hydrolysis	Instrument: Dionex DX‐500 Column: PA 10 Elution: gradient ^a^ Waveform 5 ^a^	1–50 mg/L	(Suksom et al. 2015)

Abbreviations: Ara = arabinose, Fru = fructose, Fuc = fucose, Gal = galactose, Glc = glucose, Iml = isomaltose, Lac = lactose, Mal = maltose, Mal3 = maltotriose, Rha = rhamnose, Rib = ribose, Suc = sucrose, Xyl = xylose.

^a^ Programs and waveforms in Supporting Information.

Both Mechelke et al. (2017) and Villacís‐Chiriboga et al. (2023) studied monosaccharide composition of prebiotic polysaccharides but used different hydrolysis techniques. Mechelke et al. (2017) used multiple enzymes including xylosidase, three xylanases, arabinoxylanase, cellulase, endo‐β‐1,4‐glucanase, arabinofuranosidase and ACCELLERASE® BG for full hydrolysis of any remaining polysaccharides. This is in contrast to Villacís‐Chiriboga et al. (2023) who used a sulfuric acid hydrolysis method.

Similar to the study of fruit skins done by Villacís‐Chiriboga et al. (2023), Marques et al. (2022) looked at the cell wall components of the strawberry tree ( *Arbutus unedo* ) fruits using a two‐step acid hydrolysis and found that glucose, xylose, and arabinose were the most abundant monosaccharides. Another study that applied enzyme hydrolysis was Lin et al. (2018) in which β‐galactosidase was used for the hydrolysis of terminal galactose from galactooligosaccharides.

Suksom et al. (2015) investigated sucrose degradation within the refining process of raw sugar to white sugar. They analyzed free saccharides (sucrose) before hydrolysis and again after hydrolysis into its constituent glucose and fructose which resulted in a double‐check method for sucrose content. In a further demonstration of the advantage of the IC/PAD for saccharide analysis, Pico et al. (2015), using α‐amylase and α‐amyloglucosidase for hydrolysis, claimed to be the first to separate and report isomaltose concentrations in flour.

### 4.5 Measurement Uncertainty and Limits of Detection for IC/PAD Analysis

Measurement uncertainty (MU) varied across the IC/PAD literature (Tables 2 and 3), with most studies either not reporting or not explaining how they calculated MU. Among the authors that did report MU, the majority used relative standard deviations (RSDs) with Zhang, Zhu, et al. (2022), de Souza et al. (2019), Ni et al. (2016) and Akšić, Tosti, et al. (2019) using the results of three injections. Gangola et al. (2014), Vennard et al. (2020), and Xiao et al. (2022) used RSDs but did not clearly state how many replicate injections were considered.

Based on the Horwitz curve (the effect of concentration on reproducibility), larger replicate sizes are likely to provide more accurate reflections of MU. Pico et al. (2015) used the results of nine injections, Suksom et al. (2015) used 10, while Shaaban et al. (2021) used 20 replicates. Monti et al. (2019) used RSDs of five and 44 replicates. Unlike the rest of the reported literature, Fa et al. (2017) used spike recoveries for MU calculations, whereas Hungerford et al. (2021) and Zhang, Hungerford, et al. (2022) used MUs calculated from the analysis of external reference materials.

The values presented as calibration ranges in Tables 2 and 3, cover the concentration range of the analytes on the instrument used for each study. Instrumental limits of detection (LODs) (typically calculated as three‐time analyte signal to background signal) and are expected to be at least an order of magnitude below the bottom point of each calibration range. 20 μg/L was the most common lower instrument limit of detection, with some studies reporting as low as 0.4 μg/L. In contrast method LODs, which are higher than instrument LODs, as corrections for sample dilution and sample weight have been applied. Method LODs for the literature (Tables 2 and 3), either reported for calculated, ranged from 0.25 mg/L in wine (Fa et al. 2017) to 8 mg/kg in chickpeas (Gangola et al. 2014). These figures are comparable with calculated method LODs for other forms of instrumentation use for contemporary studies, such as 85 mg/kg in 107 Korean food products using trimethylsilyl‐oxime (TMSO) derivatives by GC/FID (Jeong et al. 2023) and 2.2 g/kg of mayonnaise using HPLC/RID (Tiwari et al. 2023).

## 5 Analysis of Simple Saccharides by IC/MS—An Emerging Area

**FIGURE 5:**
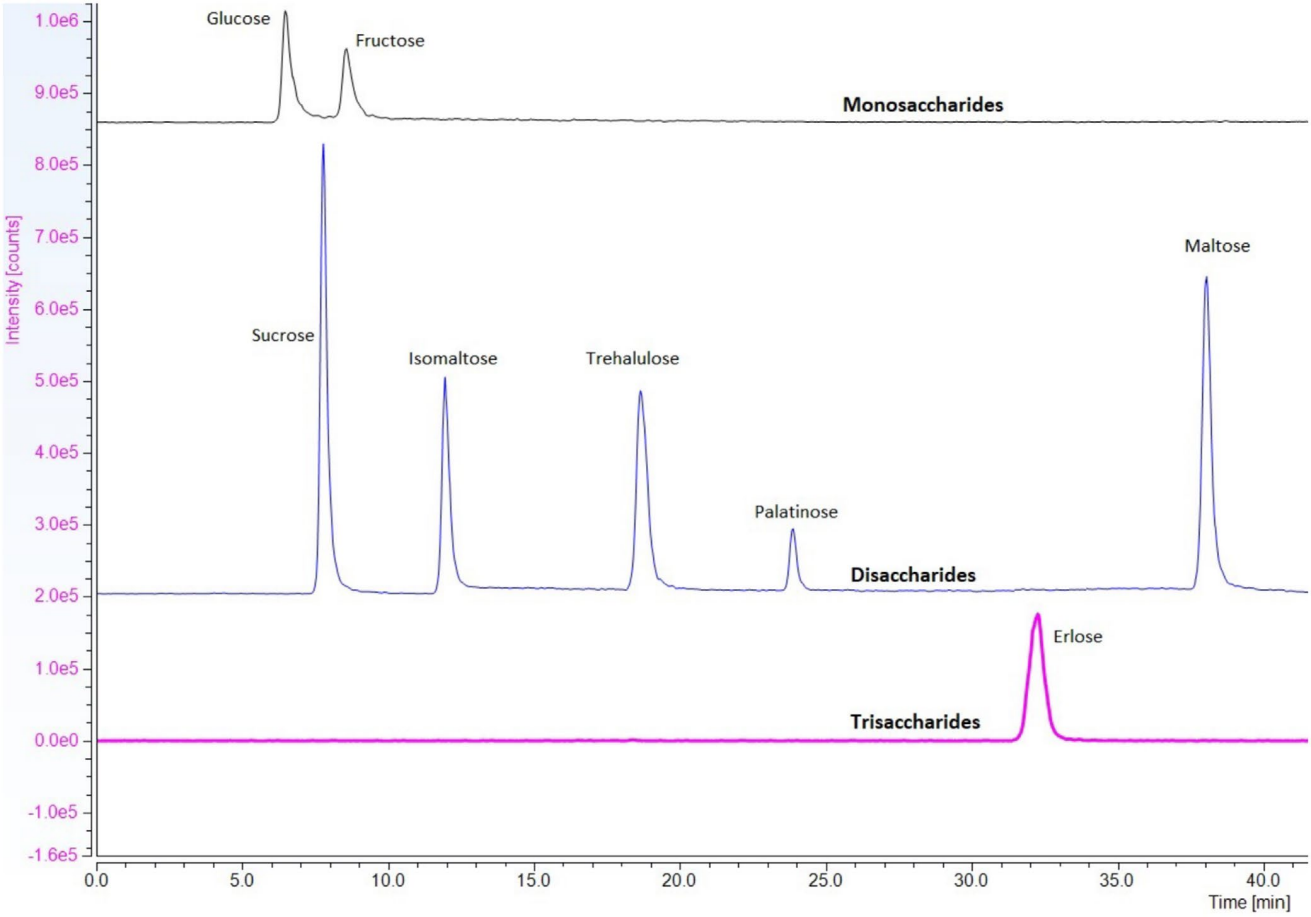
Example overlayed chromatograms showing molecular adduct ion peaks of eight saccharides. Masses were determined using the [M + Cl]^−^ ion, with specific masses for monosaccharides as 215 m/z, disaccharides as 377 m/z and trisaccharide as 539 m/z.

The first reported use of IC/MS for the analysis of saccharides was by Conboy and Henion in 1992 (Conboy and Henion 1992) while modern IC/MS methods for analysis of saccharides were described in detail by Bruggink et al. (2005). MS detection provides confirmation of peak identity using mass to charge (*m/z*) ratio (Figure 5) rather than just retention time (Figure 4). Fragment ions produced by collision‐induced dissociation of larger DP saccharide molecules provided further confirmation of the identity of the saccharides (Tian et al. 2023). Unlike HPLC, MS is not commonly combined with IC due to incompatibility with the hydroxide in the mobile phase. Successful coupling IC to MS requires use of a suppressor, previously referred to as a desalter (Bruggink et al. 2005). The suppressor neutralizes the hydroxide in the mobile phase to water through a process of electrically powered ion osmosis, making the eluant compatible with MS. In comparison to the amount of saccharide analysis studies performed using an IC/PAD in the past decade, there is less literature available for IC/MS (comparing Tables 2, 3, 4), most likely due to the additional requirements and complexity of operating an IC/MS. All the literature reviewed here used electrospray ionization. Atmospheric pressure chemical ionization (APCI) is not compatible with IC due to the non‐volatile nature of saccharides (Kurzyna‐Szklarek et al. 2022).

**TABLE 4:** Summary of data on analysis of sugars by IC/MS in food during the last decade.

Samples	Analytes	Ion chromatography		Make‐up		Mass spectrometry			References
		Column	Suppressor (mA)	Reagent	Flow rate mL/min	MS	Mode	Source voltage (kV) and temperature	
Artichoke pectin	Ara, Fru, Gal, Glc, Mal, Man, Rha, Xyl	PA1	ASRS 300 @ 75 mA	50 mM NH_4_Ac in IPA	0.003	IT	+	5 @ 275°C	(Xu et al. 2014)
Beans	Gal, Glc, Fru, Suc, Lac, Cel, Mal	PA20	ND	ND	ND	Q	ND	ND	(John and Luthria 2015)
Grape marc	Oligosaccharides	PA300	ERD 500 @ 500 mA	NU	NU	OT	−	3.2 @ 320°C	(Tian et al. 2023)
Dairy products	Gal, Glc, Lac, lactulose	PA20	AERS 500	ND	ND	OT	−	3.5 @ 280°C	(Panseri et al. 2021)
Coffee beans	Ara, Gal, Glc, Suc, Xyl, Man, Fru, Rib	PA20	AERS 500 @120 mA	0.25 mM NaAc in 2.4 M ACN	0.01	QQQ	+	4.5 @ ND	(Zhang et al. 2017)
Milk and infant formula	Gal, Glc, Suc, Lac	PA1	ASRS 300 @ 62 mA	ND	ND	QQQ	−	2.6 @350	(Wu et al. 2020)
Honey	Ara, Erl, Fru, Gal, Glc, Im3, Koj, Lac, lactulose, Man, Mel, Mlz, Nig, Pal, Raf, Rib, Suc, Tur, Xyl	PA10	ASRS 500	7 mM NH_4_OH in MeOH	0.025	Q	−	2–3 @ 400°C	(Tedesco et al. 2020)
Milk	3 Sll, 6 Sll, Lac4	PA300	ERS 500 @ 250 mA	NU	NU	OT	ND	3.2 @ 320°C	(Rumachik et al. 2021)
Spirulina	Ara, Fru, Fuc, Gal, Glc, Man, Rha, Rib, Suc, Xyl	PA20	CMD @ 212 mA	1 mM NaAc in ACN	0.2	Q	Both	500°C	(Zhao et al. 2017)
Stingless bee honey	Trehalulose	PA210	ADRS 600	ND	ND	ND	ND	ND	(Yates et al. 2025)
Pea pods	Glc, Fru, Suc	PA1	AERS 500 @ 65 mA	NU	NU	QQQ	−	3.2 @ 320°C	(Fichtner et al. 2017)
Plant tissue	Ara, Lyx, Rib, Xyl, Cel, Imu, Lac, Lam, Mal, Mel, Suc, Tur, Tre, 1Ks. Raf, sugar alcohols	PA10	AERS 500 @ 65 mA	NU	NU	QQQ	−	3.5 @ 400°C	(Feil and Lunn 2018)
	All, Alt, Fru, Fuc, Gal, Glc, Gul, Man, Psi, Rhm, Sor, Tag, Tal	SA10	AERS 500 @ 5 mA	NU	NU	QQQ	−	3.5 @ 400°C	(Feil and Lunn 2018)

Abbreviations: 1Ks = 1‐Kestose, 3 Sll = 3'‐Sialyllactose, 6 Sll = 6'‐Sialyllactose, Ara = arabinose, Cel = cellobiose, Cel3 = cellotriose, Erl = erlose, Fru = fructose, Fuc = fucose, Gal = galactose, Glc = glucose, Iml3 = isomaltotriose, Imu = Isomaltulose, IT = ion trap, Koj = kojibiose, Lac = lactose, Lac4 = lacto‐N‐neotetraose, Lam = laminarbiose, LIT = linear ion trap, Mal = maltose, Man = mannose, Mel = melibiose, Mlz = melezitose, ND = not described, NU = not used, OT = orbitrap, Pal = palatinose, Q = single quadrupole, QQQ = triple quadrupole, Raf = raffinose, Rha = rhamnose, Rib = ribose, Suc = sucrose, TOF = time of flight, Tre = trehalose, Tur = turanose, Xyl = xylose, Xyl2 = xylobiose, Xyl3 = xylotriose.

The most common use of IC/MS (Table 4) for saccharide determination is the analysis of mono‐ and disaccharides in retail foods. These sample types include: coffee (Zhao et al. 2017), infant formula, pure, fermented and ultra‐high temperature processed milk as well as yogurt, butter, and cheese (Panseri et al. 2021; Wu et al. 2020) and honeys (Tedesco et al. 2020). Three milk‐based oligosaccharides, reported to have prebiotic characteristics, were analyzed in lactose‐free milk by Rumachik et al. (2021). Other methods analyzed edible agricultural products including chardonnay grape marc (the leftovers from wine making) shown to contain a variety of beneficial bioactive compounds (Tian et al. 2023). Saccharides within pea plants and plant tissue were also studied by both Fichtner et al. (2017) and Feil and Lunn (2018). Three varieties of beans (black, dark red, and Borlotti) were investigated by John and Luthria (2015). Only two studies have used hydrolysis to examine bound sugars components, one in artichoke pectin (Xu et al. 2014) and another in spirulina powder, bought from local markets and retail stores (Zhao et al. 2017).

Each of these studies (Table 4) used different instrument configurations including flow rates, columns and MS technologies. Commonalities between the methods included IC instruments (ICS 5000), suppressors (AERS 500 or similar), MS polarity (mostly negative), target quasimolecular ions (either [M + H]^+^ or [M ‐ H]^−^), source voltage (3–3.5 kV), and scan types (SIM or MRM). Only one study used lithium adducts and two used sodium adducts. The use of these non‐volatile salts should be carefully considered, as deposits can build up in the MS interface (Sargent 2013).

Use of SPE for sample preparation occurred sporadically across studies, with Tian et al. (2023) using a sequence of porous graphitized carbon and C18 cartridges. Rumachik et al. (2021) only used the porous graphitized carbon. Fichtner et al. (2017) reported using a multiscreen‐PCR_96_ filterplate for the removal of high molecular weight contaminants. Most other studies used centrifugation as the main separation technique. Both Zhao et al. (2017) and Wu et al. (2020) used both centrifugation and C18 SPE cartridges for sample preparation and Tedesco et al. (2020) just diluted honeys studied prior to analysis.

These studies demonstrated advances in IC column technology. Commercial IC columns are constructed from divinylbenzene resin microbeads to which different functional groups are attached. Rohrer (2021) states that analysis time for IC can be reduced by employing smaller columns and smaller internal particle sizes. Smaller columns also reduce eluent and sample injection volumes, and help provide for more efficient suppression of background ions from target ions. Columns are available in many internal diameters, from capillary to 4 mm and typically two lengths 150 or 250 mm (Rohrer 2021). In terms of stationary phase, the oldest CarboPac PA1 (from 1983), is not mechanically stable, resulting in long analysis times due to low flow rates (Michalski and Kończyk 2024). Advances of smaller particle sizes with more stable and higher efficiency resins have occurred since. The PA1 phase was replaced by PA10 for simple saccharides and PA100 for oligosaccharides, which itself was replaced with PA200 (Michalski and Kończyk 2024). Table S1 displays more information with regards to column times, dates introduced, and stationary phases used in the studies presented in Tables 2, 3, 4. Two studies have showcased the newest column formats. Zhao et al. (2017) demonstrating the higher analysis speed and efficiency of the CarboPac PA20 over PA10. This is because PA20 has a smaller diameter ethylvinylbenzene and divinylbenzene microbead substrate (6.5 μm), as opposed to the PA10 (10 μm) (Michalski and Kończyk 2024). The CarboPac PA300 (4 μm), which was launched during the last decade (2020), was shown to be more efficient than the PA200 (8.5 μm) by Rumachik et al. (2021).

IC/MS methodologies can be divided into those with and without adduct formation, with the former enhancing detection sensitivity. Of those using adduct formation to enhance detection sensitivity, the most common make up phase was an acetate salt in a non‐aqueous solvent. Xu et al. (2014) used an optimized sheath liquid containing ammonium acetate in isopropanol to create [M + NH_4_]^+^ adducts. Zhao et al. (2017) used sodium acetate in acetonitrile to create [M + Na]^+^ adducts for analytes galactose, glucose, mannose, fructose and sucrose. Curiously though, the remainder of the analytes in this study were reported as either [M + H]^+^ or [M ‐ H]^−^ ions, with little comparison given to show why the adduct ions [M + Na]^+^ were not continued for each of the analytes. John and Luthria (2015) do not state which make up phase was used, only that the saccharides were observed as [M + Li]^+^ adducts. Methods not using adduct formation typically used higher resolution or more selective mass spectrometers. Tian et al. (2023), Panseri et al. (2021) and Rumachik et al. (2021) all reported using orbitrap instruments, whereas, Fichtner et al. (2017), Feil and Lunn (2018), Wu et al. (2020) reported using triple quadrupole instruments. Only Zhang et al. (2017) reported using [M + Na]^+^ adducts from sodium acetate in mixed acetonitrile and water, using a triple quadrupole. Tedesco et al. (2020) used deprotonated molecular ions [M ‐ H]^−^ ion for detection promoted by applying a post‐column infusion of ammonium hydroxide in methanol. As this occurred after the suppressor, it reintroduced hydroxide ions into the mobile phase, which are incompatible with MS and negates the suppressor removing them.

To demonstrate the selectivity of IC, John and Luthria (2015) compared it to a GC/MS method for the analysis of sugars from beans and identified two additional analytes not observed by GC/MS. These were identified as cellobiose and a further disaccharide, which was unable to be identified by retention time alone with available standards. Hence, a continuing theme in IC is that more analytes are reported than can be identified by retention time matching with reference materials. This problem appears to have been solved in part by Yates et al. (2025), who was able to demonstrate fraction collection using a modified IC/MS. The example compound used was trehalulose, a rare sugar characteristic of stingless bee honey. The compound was concentrated and its identity confirmed by both IC/PAD and nuclear magnetic resonance characterization.

LODs from IC/MS methods were comparable with equivalent HPLC/MS studies of the same sample types. Wu et al. (2020) and Panseri et al. (2021) demonstrated method LODs of 1 mg/L for lactose in milk compared to a study using HPLC/MS/MS, which reported 15 mg/L (Ohlsson et al. 2017). Tedesco et al. (2020) reported method LODs of 50–70 μg/kg for glucose, galactose, sucrose, mannose, and ribose in honey using a IC single quadrupole MS, whereas Kozłowicz et al. (2020) reported method LODs of 2 mg/kg for glucose, fructose, and sucrose by GC/MS/MS. Consistent approaches to measurement uncertainly were observed, with replication being the main method. The number of replicates varied from three by Zhao et al. (2017) to five by Tedesco et al. (2020) per method. Zhao et al. (2017) compared the sample method on IC/PAD to IC/MS for the nine monosaccharides and one disaccharide and found an order of magnitude difference between the two detectors, finding the PAD to be more sensitive.

## 6 Future Directions of IC/PAD and IC/MS Applications in Saccharide Analysis

While a wide variety of food types are presented in this review, they by no means, cover all possible foods. Processed and complex food samples, such as bakery items, confectionery, or non‐alcoholic drinks are not represented at all, while some unprocessed food samples covered such as vegetables (only potato and sweet corn) lack coverage. New, novel (edible insects, hemp seed etc.) and admixed foods (sandwiches, ready meals, etc.) need to be investigated to demonstrate the suitability of IC based techniques. Only by applying IC/PAD and IC/MS to these more complex, types of analysis can a true understanding of its strengths and weakness be fully realized. Likewise, there is still a need to investigate the presence of less common saccharides within most of the sample types mentioned in this review since the most common saccharides (e.g., glucose, fructose, and sucrose) do not represent all saccharides present in each food sample type (Amoah et al. 2023).

Additionally, there is a growing need for consistent instrument methodology across food types. This includes sample preparation and extraction, instrumentation and elution gradients. In terms of IC/MS analysis this also includes standardization of the suppressor, suppressor program, makeup solvent, and MS mode across all analyses. Columns and MS types will doubtless change, depending on the target compounds of the analysis (e.g., detection of mono‐, di‐, oligo‐, or polysaccharides).

While an effort by the AOAC to form a consistent IC/PAD method for dairy products (including sample preparation and instrument program) is presented by Brunt et al. (2021) and Sanders et al. (2019), the adoption of such a method would only cover specific products (Brunt et al. 2021; Sanders et al. 2019). Therefore, there is a pressing need for method standardization using other sample types (e.g., cereal products, sugar products) by both researchers and organizations overseeing global analysis methodology (e.g., AOAC, IHC). These methodologies need to include instrumentation, sample preparation, and extraction. To date, the limited sample types, limited analytes, as well as narrow reasons for analysis, provide the largest opportunities for further analysis using this technique.

It is therefore suggested that the best way forward would be to gather and create a group of like‐minded scientists and researchers, in the field of IC carbohydrate analysis. The aims of the group would be to assess both analytical methodology and the accuracy of saccharide analysis. Such a group can then start the process of reviewing, testing and updating methodologies for saccharide analysis by IC/PAD and IC/MS for food and food sample types. The methodologies can then be verified, though the use of matrix‐specific inter laboratory reference materials. Once verified, these methodologies can be published and promoted for adoption.

## 7 Conclusion

This review focused on DP 1–3 saccharides present in foods, which are referred to as sugars for regulatory purposes. Several natural and technological production processes can impact the concentration of saccharides present in food at the time of consumption, causing variability in both the quantity and nature of the saccharides present. The concentration of the saccharides present has an effect not only on the functionality and perception of the food itself, but also on the health benefits to the consumer.

Older saccharide analysis methodology had poor specificity, confounding accurate reporting. Of the contemporary analysis methodologies discussed, IC provides the best selectivity and specificity, requiring less sample preparation, and no derivatization. The studies for both IC/PAD and IC/MS covered a variety of food sample types including cereals, dairy products, honeys, as well as fruits and vegetables.

We conclude that the three main objectives for saccharide analysis by IC over the next decade should be (a) the investigation of new, novel, and admixed foods, so that the information is up‐to‐date, timely, and relevant, (b) increased utilization of IC/MS for analysis to improve separation and resolution of saccharides present, and (c) standardization of the IC methodology across food analysis so that results are easily comparable.

## Author Contributions

**Hans S. A. Yates:** conceptualization (lead), investigation (lead), methodology (lead), visualization (lead), writing – original draft (lead), writing – review and editing (equal). **James. F. Carter:** supervision (equal), writing – review and editing (equal). **Achamma Joseph:** investigation (supporting), writing – original draft (supporting), writing – review and editing (equal). **Melody Chaussende:** investigation (supporting), writing – original draft (supporting), writing – review and editing (equal). **Mary T. Fletcher:** supervision (equal), writing – review and editing (equal). **Viviene Santiago:** supervision (equal), writing – review and editing (equal). **Natasha L. Hungerford:** supervision (equal), writing – review and editing (equal).

## Ethics Statement

This study does not involve any animal or human testing.

## Conflicts of Interest

The authors declare no conflicts of interest.

## Supporting information


**Data S1:** fsn370855‐sup‐0001‐DataS1.xlsx.

## Data Availability

Data available in article Supporting Information.
